# Fulfilling the promise of digital health interventions (DHI) to promote women’s sexual, reproductive and mental health in the aftermath of COVID-19

**DOI:** 10.1186/s12978-021-01168-x

**Published:** 2021-06-04

**Authors:** Vijay Kumar Chattu, Claudia Abreu Lopes, Sumbal Javed, Sanni Yaya

**Affiliations:** 1 Department of Medicine, Temerty Faculty of Medicine, University of Toronto, Toronto, ON M5G 2C4 Canada; 2 Division of Occupational Medicine, Occupational Medicine Clinic, St. Michael’s Hospital, Unity Health Toronto, Toronto, ON M5C 2C5 Canada; 3 International Institute for Global Health, United Nations University, Kuala Lumpur, Malaysia; 4 School of Public Health, The University of Hong Kong, Pok Fu Lam, Hong Kong; 5 School of International Development and Global Studies, Faculty of Social Sciences, University of Ottawa, Ottawa, ON Canada; 6 The George Institute for Global Health, Imperial College London, London, UK

## Introduction

Globally, over 800 women die every day in pregnancy and childbirth; violence against women remains devastatingly pervasive, affecting 1 in 3 women in their lifetime, and depression rates are twice that of men, according to the World Health Organization (WHO) [1]. The report further emphasizes that sexual and reproductive health (SRH) services are quickly disrupted when health systems are under pressure which is dangerous and disempowering. Therefore, access to contraception, safe abortion to the maximum extent permitted by law, STI prevention and recovery, care and assistance for abuse survivors, and self-care interventions should all be prioritized in countries' COVID-19 responses, according to WHO [2]. As the COVID-19 pandemic paralyzes the health systems across nations, there is a significant drop in access to routine healthcare, and many patients are showing interest and turning towards telehealth, telemedicine, or remote virtual health services to access essential primary care. For example, in the United States, all the states have expanded the telehealth policies to reduce the pressure on the hospitals treating COVID-19 patients and reduce patients’ exposure [3]. Global health emergencies in the past have revealed that during the crisis, access to safe abortion can be negatively affected [4]. While countries are still grappling with COVID-19 and its response is ever-evolving. The increased burden on health systems can result in reduced access to abortion facilities. As the health systems come under mounting pressure and providers become infected, some countries have had to close down clinics offering abortion services. Such circumstances necessitate innovative solutions not only in remote places or countries with limited resources but also in developed countries [5].

During global health emergencies, there is a total reversal of priorities and, as a result, the availability, accessibility, and affordability of SRH services may become challenging, especially in resource-poor settings A study from South Africa by Pattison et al. on the impact of the first wave of COVID-19 on maternal and reproductive health services and maternal mortality showed that there had been an increase of 30% in maternal deaths since lockdown started and the pandemic peaked in 2020, compared with the same period in 2019. Use of reproductive health services (contraception and termination of pregnancy) has declined sharply since lockdown. Rural provinces are experiencing increased pressure on their services due to pregnant women migrating from metropolitan areas back to their homes, increasing the burden on already under-resourced facilities [6].

Hence, it is critical to provide effective health facilities and health delivery systems to achieve the UN SDG target 3.7 and universal access to SRHR services [7]. Sexual and reproductive health and rights (SRHR) and bodily autonomy are explicitly recognized in International human rights law. Under these rights, states are obligated to ensure access to abortion services and remove any obstacles that deny access [8]. Provision of digital health interventions (DHIs), e.g., telemedicine, mHealth, etc., is the perfect strategy for thinking innovatively to transform the existing health systems and improve the SRH services and healthcare for both the short term and long term. This technology-driven services address equity, especially for the rural communities and marginalized groups with poor access to family planning providers and specialists in Obstetrics and Gynecology. As highlighted by McCoy et al., DHI may provide access to disadvantaged or difficult-to-reach groups identified by geography, stigmatized attitudes or personalities, or people who value confidentiality. In addition, DHI connects women to contraceptives, expands HIV self-testing and HIV pre-exposure prophylaxis (PrEP) access among vulnerable groups such as men who have sex with men (MSM), and can help spread the word about low-cost maternal care services [9]. Telehealth services such as digital communication channels have a wider scope and play a significant role in sending the message to the target groups or individuals, thereby improving the service delivery of SRHR. As highlighted by Bacchus et al., digital health technologies provide both opportunities to advance the SRH and pose potential risks due to confidentiality and SRH being a highly sensitive area [10]. Through telemedicine, the existing geographic or social, or behavioral barriers in accessing the SRH services can be addressed by facilitating the self-use of these services adapted to the types and access to technology and the digital literacy skills of users [11].

## Digital health and COVID-19

In May 2018, the Seventy-First World Health Assembly (WHA) passed Resolution WHA71.7 on Digital Health to promote healthy lives and wellbeing for everyone, everywhere, at all ages. The concept includes a range of functions for promoting the Sustainable Development Goals, equitable and universal access to quality health services; increasing health systems sustainability, accessibility, and affordability; strengthening health promotion, disease prevention, treatment, rehabilitation, and palliative care. It defines Digital Health as “the field of knowledge and practice associated with any aspect of adopting digital technologies to improve health, from inception to operation” and encompasses eHealth [12]. On March 6, 2019, the WHO Director-General announced the creation of the Department of Digital Health “to enhance WHO’s role in assessing digital technologies and support Member States in prioritizing, integrating and regulating them” [13]. Digital health solutions are gaining popularity and attention and likely will persist COVID-19 pandemic revamping healthcare systems globally. The technology is improving day by day, and many developed nations have already conducted feasibility studies and implemented use in various specializations for delivering healthcare services to remote patients [14].

## Impact of COVID-19 on sexual and reproductive health and rights (SRHR)

COVID-19 has accelerated the use of digital technologies for immediate outbreak responses (including health communication, contact tracing, testing, surveillance, diagnostics, and treatment) and impact mitigation measures (including wellbeing and mental health promotion, telemedicine, support for gender-based violence survivors, financial protection). Of 96 countries surveyed by WHO, 60 have deployed telemedicine to replace in-person consultations, and many have been using a range of digital technologies in their COVID-19 responses [15]. During COVID-19, to avoid preventable complications associated with abortion, it is necessary to enable self-managed abortion through telemedicine counseling, guaranteeing access to medications, and ensuring that women are not criminalized for inducing self-abortions, a vital step towards fulfilling states’ blinding human rights obligations [5, 16]. It is encouraging that several organizations have moved their services online and continued to sustain SRHR advocacy through innovative approaches such as telemedicine, mHealth services or by partnering with other sectors such as commercial service deliveries and online commercial platforms, pushing governments to leverage the potential use of telemedicine for SRH, particularly for abortion [17].

In a pandemic, pregnancy and childbirth are not placed on hold. Whatever the circumstances, all women have the right to a healthy and supportive pregnancy and childbirth experience, and they need high-quality, compassionate, and respectful maternity care. Evidence of unnecessarily separating mothers from their newborn babies during the pandemic is also alarming, posing serious health and well-being risks [2].

Abortion is a time-sensitive service; delays can lead to unsafe abortions, restrictions on abortions or the lack of availability can turn people towards unsafe options to end a pregnancy. Several countries have enabled telemedicine for SRH services, including abortion. The United Kingdom, France, and Ireland have approved telemedicine and remote support of abortions. In addition, Albania has enabled telemedicine for prenatal care, Belgium is using telemedicine for abortion pre-meetings and prescriptions. It is in accordance with the WHO guidance, which confirms that self-managed abortion is safe, given that pregnant individuals have been fully informed on protocols and, if needed, have access to follow-up healthcare [3, 17].

Globally, numerous DHIs are targeting a range of populations for a variety of SRHR topics across continents in different cultural contexts, which have been shown to be acceptable and feasible to implement by the end-user. Some of the recent successful interventions are summarized below (Table 1).

**Table 1:** Application of various Digital Health Interventions (DHIs) in Sexual and Reproductive Health (SRH)

S. no.	Areas of improvement	Author, year, and references
1	Telemedicine for medical abortion (systematic review)	Endler, 2019 [18]
2	Online testing for sexually transmitted infections (STIs) have shown to double the update of STI tests	Wilson, 2017 [19]
3	e- contraception through which the oral contraceptive can be ordered online	Rezel, 2017 [20]
4	Improvement in levels of knowledge, contraceptive use, and health-seeking behavior (using digital innovations, mobile technologies, and interventions)	Daher,2017 [21]
5	Application of digital innovations for HIV and STIs (systematic review)	Daher, 2017 [21]
6	Expanding the choice in accessing contraception online (improving access to various socioeconomic groups)	Rezel, 2017 [20]
7	mHealth SRH interventions among female sex workers (high-risk groups)	Ampt, 2017 [22]
8	Supporting post-abortion contraception among women using mobile phone-based intervention	Smith, 2017 [23]
9	Mobile phone interventions for adolescent SRH (systematic review)	L’Engle, 2016 [24]
10	Promoting safer sex behaviors through mobile phone texting interventions	French, 2016 [25]
11	Increase in safer sex behaviors among young people using text messages	Free, 2016 [26]
12	Improvement in levels of knowledge, contraceptive use, and health-seeking behavior through digital innovations and mobile technologies	Sondaal, 2016 [27] Burns, 2016 [28] Smith, 2015 [29]

Therefore, the application of successful DHIs shows great promise in the area of SRH, which can address the issues of equity, access, and affordability, especially in certain remote settings. In this context, Crawford et al. have proposed a Digital Health Equity (DHE) framework which can be used to consider the health equity factors, and they further argued that along with person-centered care, DHE should be integrated into health provider education and promoted at the individual, institutional, and social levels [30].

However, under the guise of the COVID-19 pandemic, some governments undermine women’s health when it needs the most protection, such as Poland and Romania [17]. Some law and policymakers in the United States (US) have been effectively working to ban abortions by categorizing them as not “medically necessary” care and “non-essential.” Both US and Netherlands courts' responses towards petitions for safeguarding abortion access during this pandemic have been mixed [31]. Further, some countries have taken regressive approaches towards women’s SRR; for instance, the Lithuanian health minister has asked women to rethink abortion during their time in lockdown [17]. To cite some success stories from Africa, Zimbabwe and Nigeria had ensured continuity of SRH services by integrating them with other essential services such as immunization and food delivery programs. In Uganda, a mobile app “SafeBoda” allowed women to order contraception to their doorstep through a motorcycle [32].

Access to safe abortions is essential now more than ever; reports have indicated that states’ COVID-19 responses could increase unwanted pregnancies due to lockdowns, lack of access to contraceptive supplies, raising incidence of domestic violence, and increasing income insecurity [33]. Compelling women to continue with an unwanted pregnancy is a human rights violation under several circumstances, including foreseeable mental and physical health impacts on the pregnant person. During the COVID-19 pandemic, several health care services may be disrupted or are inaccessible due to increased burden on healthcare systems, further creating barriers to services required for a pregnant person [5, 16, 34].

## Women and mental health

Common disorders such as depression, anxiety, and somatic complaints are more common among women and affect 1 in 3 people in the community, which is a huge public health problem. Depressive disorders account for 42% of disability from neuropsychiatric disorders among women compared to 29% among men. The lifetime prevalence rate of violence against women ranges from 16–50%, and 80% of the 50 million displaced people affected by violent conflicts, civil wars, disasters, and displacements are women and children [35]. Regardless of exposure to the virus, people may experience fear and anxiety of becoming sick or dying and helpless. Some of them may blame other people who are ill, potentially triggering off a mental breakdown. There is a wide range of psychiatric morbidities that have been found, such as depression, anxiety, panic attacks, somatic symptoms, and posttraumatic stress disorder (PTSD) symptoms, to delirium, psychosis, and even suicidality [36]. A study of COVID-19 and adverse mental health outcomes by Gold et al. highlighted that healthcare workers, 70% of which are women, are at a high risk of mental health problems [37].

Anxiety and/or depressive disorders affect up to 20% of those seeking primary health care in developing countries, and many health professionals have gender biases that cause them to either over-treat or under-treat women when they dare to report their problems. Therefore, the WHO [35] emphasizes on 3 key areas to address women’s mental health, namely:

1. Build evidence on the prevalence, causes, mediating factors, and protective factors for mental health problems among women.
2. Encourage the formulation and implementation of health policies that address the needs and concerns of women from childhood to old age.
3. Improve primary care providers' ability to recognize and manage the mental health effects of domestic violence, sexual harassment, and acute and chronic stress in women.

## Application of digital health interventions (DHIs) in mental health

Mental health support to frontline health workers, patients, and carers will be crucial, as long isolation, lack of social interaction, as well as anxiety over one’s own and others’ health will take a toll on well-being [38]. Psychiatrists, psychotherapists, and psychologists need to ensure that they are maintaining their own mental health during this time, with programs such as professional supervision being of help [39]. Telemedicine services will become increasingly crucial in the pandemic setting, as physical isolation and frontline work pose both access issues and mental health stressors [40]. The various studies done among diverse groups of patients to assess different digital mental health interventions are summarized below (Table 2).

**Table 2:** Various digital health interventions to address mental health issues

Author, year- Country, References	Target Group	Conditions of interest	Digital health intervention	Outcomes
Bhaskar and Bradley et al. (2020)—Multi-country consortium—Australia [39]	Stroke patients	Mental health and functional abilities	Telerehabilitation programs (involving consultations, exercises, games, and therapy aspects)	Positive outcomes such as improving patients’ functional abilities and mental health
van Houwelingen CT et al. 2018—Netherlands [41]	Elderly patients with diagnosed mental health conditions	All general health issues, including mental health	Telehealth—intention to use videoconferencing and capacities to use digital technology	Self-efficacy and digital literacy presumably have a significant impact on the uptake of telehealth among the elderly
Bhaskar et al. 2020—Part 2 Multi-country consortium, Australia [42]	Caregivers and family members of patients with neurological conditions	The worried family members who have voiced concerns that physically distanced visits such as through windows may further confuse their loved ones	Telemedicine	Telemedicine has been utilized to connect to prevent further decline in mental status and provide comfort
Chan 2017—USA [43]	General Psychiatric patients (Review of studies)	General mental health illnesses delivered via smartphone apps and digital outreach programs	Telepsychiatry, telemental health, mobile mental health, informatics, cellular phone, ambulatory monitoring, telemetry, and algorithms	Psychoeducation and mental well-being advice can be leveraged
Nemecek 2019—Austria [44]	Oncology patients and caregivers	Anxiety and Depression	Telemedicine	Significant reduction in anxiety and depression levels in the telemedicine group vs. the standard care
Ruskin 2004—USA [45]	Patients of Depression	Treating Depression in-person vs. telepsychiatry	Telepsychiatry	Equivalent levels of patient adherence, patient satisfaction, and healthcare cost
O’Reilly et al. 2007—Canada [46]	Psychiatric patients (Randomized control trial)	All Psychiatric conditions	Telepsychiatry	Psychiatric consultation and short-term follow-up provided by telepsychiatry can produce clinical outcomes equivalent to face-face Telepsychiatry was less expensive than face-to-face service Telepsychiatry may not produce equivalent outcomes when used to deliver psychotherapy, which is more dependent on the therapist-patient relationship
Salisbury et al. 2016—UK [47]	Patients with Depression	Depression	Integrated Telehealth services	Telehealth service leveraging by non-clinical health advisers supporting patients in the use of Internet resources was both acceptable and effective compared with regular care
Chipps et al. 2012—South Africa [48]	Resource-poor settings (Systematic review of evidence)	Psychiatric disorders	Videoconference based Telepsychiatry	Telepsychiatry is effective and feasible. Can be tried to integrate into local health systems based on a case by case after evaluation
Hassan 2019—USA [49]	Refugee populations (Systematic review)	Mental health disorders	Telepsychiatry (psychotherapeutic treatment via videoconferencing)	Evidence pointing towards the efficacy of telepsychiatry in resource-constrained environments Psychotherapeutic treatment delivered via video conferencing is just as effective as traditional treatment, albeit less desirable

## Protection of SRHR

The state’s obligation under international human rights law to protect, respect, and protect the right to health, life, and non-discrimination, among other rights, should not be interrupted in times of crisis. Measures should be taken to prevent unsafe abortion while ensuring that access to SRH services, including abortion, are non-derogable core obligations of states and should be upheld even during a crisis such as COVID-19 [50–52]. Therefore, to fulfill these core obligations, policies and laws that criminalize or obstruct access to sexual and reproductive services should be repealed. Governments must adopt WHO guidelines and a patient-centered, human rights-based approach. They must adapt their technical guidance, policies, and service-delivery models to guarantee access to SRHR by allowing telemedicine during the crisis [5, 17]. The resistance towards making safe abortion has highlighted the importance of including feminist methodologies in global health research to reveal both formal and informal ways by which gender inequality manifests in healthcare access and its delivery. To ensure inclusivity and representation, we must actively consider what barriers to participation exist, whose voices are missing and what methods are used to expose these factors; above all, the global health agenda must be feminist [53].

WHO has made progress on many facets of women's rights, health, and gender equality over the last 25 years, as outlined in the visionary global policy framework, the 1995 Beijing Platform for Action on Women. Supporting feminist movements that keep governments accountable and drive change in societies by using a human rights-based approach is critical for continuing to advance the health and well-being of women everywhere, in all their diversity [54].

## Conclusions

The COVID-19 pandemic has disrupted SRH services across the world, resulting in many unwanted pregnancies, stillbirths, maternal and neonatal deaths, with negative impacts on mental health outcomes for women. Despite the challenges, some countries have leveraged health technologies to ensure the access and delivery of healthcare, paving the way to a digital health future. During lockdowns, mHealth and telemedicine have been gained global prominence revealing their potential beyond serving marginalized and underserved communities. In a post-COVID era, there are also opportunities to improve healthcare access and promote gender equality. Poverty, a lack of access to digital health interventions (DHIs), a lack of engagement with digital health in some communities, and barriers to digital health literacy are some factors that can lead to poor health outcomes. Therefore, digital health equity (DHE) should be integrated into the health policies to address the issues of equity, access, and affordability, especially in remote settings. Hence, there is an urgent call for health systems to be intentional in correcting broader gender inequities and in integrating digital health technologies to build resilience to future health crises.

## Data Availability

Not applicable.

## References

[1] WHO. Violence Against Women Prevalence Estimates, 2018. https://www.who.int/publications/i/item/9789240022256. Accessed 24 Apr 2021

[2] WHO. Six priorities for women and health. March 25, 2021. https://www.who.int/news-room/spotlight/6-priorities-for-women-and-health. Accessed 24 Apr 2021

[3] CCHPCA. Center for Connected Health Policy, “COVID-19 Related State Actions. https://www.cchpca.org/resources/covid-19-related-state-actions. Accessed 17 Oct 2020

[4] UNFPA. Rapid assessment of Ebola impact on reproductive health services and service seeking behaviour in Sierra Leone. Freetown: UNFPA. 2015. https://reliefweb.int/sites/reliefweb.int/files/resources/UNFPA%20study%20_synthesis_March%2025_final.pdf. Accessed 24 Apr 2021

[5] Todd-Gher J, Shah P (2020). Abortion in the context of COVID-19: a human rights imperative. Sex Reprod Health Matters.

[6] Pattinson R, Fawcus S, Gebhardt S, Niit R, Soma-Pillay P, Moodley J (2020). The effect of the first wave of Covid-19 on use of maternal and reproductive health services and maternal deaths in South Africa. Obstet Gynaecol Forum.

[7] Chattu VK, Yaya S (2020). Emerging infectious diseases and outbreaks: implications for women’s reproductive health and rights in resource-poor settings. Reprod Health.

[8] Center for Reproductive Rights. Breaking ground: treaty monitoring bodies on reproductive rights. New York, NY: Center for Reproductive Rights; 2020. https://reproductiverights.org/sites/default/files/documents/Breaking-Ground-2020.pdf. Accessed May 4, 2021

[9] McCoy SI, Packel L (2020). Lessons from early stage pilot studies to maximize the impact of digital health interventions for sexual and reproductive health. mHealth.

[10] Bacchus LJ, Reiss K, Church K, Colombini M, Pearson E, Naved R, Smith C, Andersen K, Free C (2019). Using digital technology for sexual and reproductive health: are programs adequately considering risk?. Glob Health.

[11] Remme M, Narasimhan M, Wilson D (2019). Self care interventions for sexual and reproductive health and rights: costs, benefits, and financing. BMJ.

[12] WHO. Global Strategy on Digital Health 2020–2024, Draft March 26, 2019. https://extranet.who.int/dataform/upload/surveys/183439/files/Draft%20Global%20Strategy%20on%20Digital%20Health.pdf. Accessed 21 Apr 2020

[13] WHO (n,a). WHO releases first guideline on digital health interventions, April 17, 2019. https://www.who.int/news/item/17-04-2019-who-releases-first-guideline-on-digital-health-interventions#:~:text=On%206%20March%202019%2C%20Dr,prioritizing%2C%20integrating%20and%20regulating%20them. Accessed 21 Apr 2020

[14] Chattu VK, Chattu SK, Adisesh A, Lal BS, Patel N (2020). Global and national digital health strategies for the post-COVID-19 era. Economics of Covid-19 digital health education & psychology.

[15] WHO. Pulse survey on continuity of essential health services during the COVID-19 pandemic: interim report, 27 August 2020. https://www.who.int/publications/i/item/WHO-2019-nCoV-EHS_continuity-survey-2020.1. Accessed 31 Mar 2020

[16] Purdy C. How will COVID-19 affect global access to contraceptives – and what can we do about it? DevEx [Internet]. 2020 Mar 11. https://www.devex.com/news/opinion-how-will-covid-19-affect-global-access-to-contraceptives-and-what-can-we-do-about-it-96745. Accessed 31 Mar 2020

[17] Hickson C, Datta N. Sexual and Reproductive Health and Rights during the COVID-19 pandemic [Internet]. European Parliamentary Forum for Sexual & Reproductive Rights and IPPF European Network; 2020. https://www.ippfen.org/sites/ippfen/files/2020-04/Sexual%20and%20Reproductive%20Health%20during%20the%20COVID-19%20pandemic.pdf. Accessed 30 Apr 2020

[18] Endler M, Lavelanet A, Cleeve A, Ganatra B, Gomperts R, GemzellDanielsson K (2019). Telemedicine for medical abortion: a systematic review. BJOG.

[19] Wilson E, Free C, Morris TP (2017). Internet-accessed sexually transmitted infection (e-STI) testing and results service: a randomized, single-blind, controlled trial. PLoS Med.

[20] Rezel E, Free C, Syred J, Baraister P. Online Contraception - Innovation to Expand Choice in Access. Poster presented at: Faculty of Sexual & Reproductive Healthcare of the Royal College of Obstetricians & Gynaecologists Annual Scientific Meeting; 27–28 April 2017; Cardiff, Wales.

[21] Daher J, Vijh R, Linthwaite B (2017). Do digital innovations for HIV and sexually transmitted infections work? Results from a systematic review (1996–2017). BMJ Open.

[22] Ampt FH, Mudogo C, Gichangi P (2017). WHISPER or SHOUT study: protocol of a cluster randomized controlled trial assessing mHealth sexual reproductive health and nutrition interventions among female sex workers in Mombasa, Kenya. BMJ Open.

[23] Smith C, Ly S, Uk V, Warnock R, Free C (2017). Women’s views and experiences of a mobile phone-based intervention to support post-abortion contraception in Cambodia. Reprod Health.

[24] L’Engle KL, Mangone ER, Parcesepe AM, Agarwal S, Ippoliti NB (2016). Mobile phone interventions for adolescent sexual and reproductive health: a systematic review. Pediatrics.

[25] French RS, McCarthy O, Baraitser P, Wellings K, Bailey JV, Free C (2016). Young people’s views and experiences of a mobile phone texting intervention to promote safer sex behavior. JMIR mHealth Uhealth.

[26] Free C, McCarthy O, French RS (2016). Can text messages increase safer sex behaviours in young people? Intervention development and pilot randomized controlled trial. Health Technol Assess.

[27] Sondaal SFV, Linda Browne J, Amoakoh-Coleman M (2016). Assessing the effect of mHealth interventions in improving maternal and neonatal care in low-and middle-income countries: a systematic review. PLoS ONE.

[28] Burns K, Keating P, Free C (2016). A systematic review of randomized control trials of sexual health interventions delivered by mobile technologies. BMC Public Health.

[29] Smith C, Gold J, Ngo TD, Sumpter C, Free C (2015). Mobile phone-based interventions for improving contraception use. Cochrane Database Syst Rev.

[30] Crawford A, Serhal E (2020). Digital health equity and COVID-19: the innovation curve cannot reinforce the social gradient of health. J Med Internet Res.

[31] Human Rights Committee. General Comment No. 36: para 26. UN Doc. CCPR/C/GC/36; 2018. https://tbinternet.ohchr.org/Treaties/CCPR/Shared%20Documents/1_Global/CCPR_C_GC_36_8785_E.pdf. Accessed 21 April, 2021

[32] UNFPA. United Nations Population Fund. Ride-hailing app delivers contraceptives to users’ doorsteps. July 17, 2020. https://www.unfpa.org/news/ride-hailing-app-delivers-contraceptives-users-doorsteps. Accessed 12 May, 2021

[33] UNFPA. Impact of the COVID-19 Pandemic on Family Planning and Ending Gender-Based Violence, Female Genital Mutilation and Child Marriage. 2020; https://www.unfpa.org/sites/default/files/resource-pdf/COVID-19_impact_brief_for_UNFPA_24_April_2020_1.pdf. Accessed 12 May, 2021

[34] Javed S, Chattu V (2021). Patriarchy at the helm of gender-based violence during COVID-19. AIMS Public Health.

[35] WHO. Mental Health and Substance Use. Gender and women’s mental health. https://www.who.int/teams/mental-health-and-substance-use/gender-and-women-s-mental-health. Accessed 24 April, 2021

[36] Toosi M, Zaki N, Chattu VK, Lal BS, Patel N (2020). Psychological and psychiatric consequences of COVID-19. Economics of covid-19 digital health education & psychology.

[37] Gold JA (2020). Covid-19: Adverse mental health outcomes for healthcare workers. BMJ.

[38] Bhaskar S, Sharma D, Walker AH, McDonald M, Huasen B, Haridas A (2020). Acute neurological care in the COVID-19 era: the pandemic health system REsilience PROGRAM(REPROGRAM) consortium pathway. Front Neurol.

[39] Bhaskar S, Bradley S, Israeli-Korn S, Menon B, Chattu VK, Thomas P (2020). Chronic neurology in COVID-19 era: clinical considerations and recommendations from the REPROGRAM consortium. Front Neurol.

[40] DeJong SM (2018). Professionalism and technology: competencies across the tele-behavioral health and E-behavioral health spectrum. Acad Psychiatry.

[41] van Houwelingen CT, Ettema RG, Antonietti MG, Kort HS (2018). Understanding older people’s readiness for receiving telehealth: mixed method study. J Med Internet Res.

[42] Bhaskar S, Bradley S, Chattu VK, Adisesh A, Nurtazina A, Kyrykbayeva S, Sakhamuri S, Moguilner S, Pandya S, Schroeder S, Banach M (2020). Telemedicine as the new outpatient clinic gone digital: position paper from the Pandemic Health System REsilience PROGRAM (REPROGRAM) International Consortium (Part 2). Front Public Health.

[43] Chan S, Godwin H, Gonzalez A, Yellowlees PM, Hilty DM (2017). Review of use and integration of mobile apps into psychiatric treatments. Curr Psychiatry Rep.

[44] Nemecek R, Huber P, Schur S, Masel EK, Baumann L, Hoeller C (2019). Telemedically augmented palliative care: Empowerment for patients with advanced cancer and their family caregivers. Wien Klin Wochenschr.

[45] Ruskin PE, Silver-Aylaian M, Kling MA, Reed SA, Bradham DD, Hebel JR (2004). Treatment outcomes in depression: comparison of remote treatment through telepsychiatry to in-person treatment. Am J Psychiatry.

[46] O’Reilly R, Bishop J, Maddox K, Hutchinson L, Fisman M, Takhar J (2007). Is telepsychiatry equivalent to face-to-face psychiatry? Results from a randomized controlled equivalence trial. Psychiatr Serv.

[47] Salisbury C, O’Cathain A, Edwards L, Thomas C, Gaunt D, Hollinghurst S (2016). Effectiveness of an integrated telehealth service for patients with depression: a pragmatic randomized controlled trial of a complex intervention. Lancet Psychiatry.

[48] Chipps J, Brysiewicz P, Mars M (2012). Effectiveness and feasibility of telepsychiatry in resource constrained environments? A systematic review of the evidence. Afr J Psychiatry.

[49] Hassan A, Sharif K (2019). Efficacy of telepsychiatry in refugee populations: a systematic review of the evidence. Cureus.

[50] UN CESCR. General Comment No. 22, para. 49 (a)(c)(e). UN Doc. E/C.12/GC/22; 2016. https://www.escr-net.org/resources/general-comment-no-22-2016-right-sexual-and-reproductive-health

[51] UN Commission on Human Rights. Siracusa Principles on the Limitation and Derogation Provisions in the International Covenant on Civil and Political Rights, Annex. UN Doc. E/CN.4/1985/4, 1984. https://undocs.org/en/E/CN.4/1985/4

[52] Sekalala S, Dagron S, Forman L, Mason MB (2020). Analyzing the human rights impact of increased digital public health surveillance during the COVID-19 crisis. Health Hum Rights J.

[53] Davies S, Harman S, Manjoo R, Tanyag M, Wenham C (2019). Why it must be a feminist global health agenda. Lancet.

[54] WHO. Improving women’s health and gender justice since the 1995 Beijing Platform for Action. https://www.who.int/initiatives/beijing25. Accessed 14 May, 2021

